# Methodological and Ethical Challenges in a Web-Based Randomized Controlled Trial of a Domestic Violence Intervention

**DOI:** 10.2196/jmir.7039

**Published:** 2017-03-28

**Authors:** Laura Tarzia, Jodie Valpied, Jane Koziol-McLain, Nancy Glass, Kelsey Hegarty

**Affiliations:** ^1^ Department of General Practice The University of Melbourne Carlton Australia; ^2^ Centre for Interdisciplinary Trauma Research Auckland University of Technology Auckland New Zealand; ^3^ School of Nursing & Bloomberg School of Public Health Johns Hopkins University Baltimore, MD United States

**Keywords:** eHealth, domestic violence, web-based trials, research design, ethics, research

## Abstract

The use of Web-based methods to deliver and evaluate interventions is growing in popularity, particularly in a health care context. They have shown particular promise in responding to sensitive or stigmatized issues such as mental health and sexually transmitted infections. In the field of domestic violence (DV), however, the idea of delivering and evaluating interventions via the Web is still relatively new. Little is known about how to successfully navigate several challenges encountered by the researchers while working in this area. This paper uses the case study of I-DECIDE, a Web-based healthy relationship tool and safety decision aid for women experiencing DV, developed in Australia. The I-DECIDE website has recently been evaluated through a randomized controlled trial, and we outline some of the methodological and ethical challenges encountered during recruitment, retention, and evaluation. We suggest that with careful consideration of these issues, randomized controlled trials can be safely conducted via the Web in this sensitive area.

## Introduction

The Internet is growing in popularity as a means of both delivering health interventions [1,2] and recruiting research participants to evaluate those interventions [3]. This is perhaps unsurprising when the advantages offered by Web-based methods are considered compared with the traditional ways of engaging and retaining participants. For instance, the Internet allows a large number of participants to be accessed, even globally if necessary, at a relatively low cost. In addition, diverse or marginalized populations can be included in a study sample where transcription errors are reduced and responses can be collected more quickly [4], both being beneficial as competition for valuable research dollars increases. Practical limitations that might have been present a few years ago—such as requiring the participants to have Internet access and a degree of computer literacy—are barely relevant in today’s increasingly networked society. In Australia, for example, household Internet access currently sits at 80% [5] and around 15 million people (80%) use a mobile phone [6]. An estimated 65% of Australians have a Facebook account [7]. Statistics for other developed countries such as the United States and the United Kingdom are similar [8,9].

Despite this potential, the use of online methods to recruit and engage participants in evaluating interventions is not without challenges. Due to the lack of face-to-face contact with researchers, online trials can experience poor retention rates, unreliable self-report data, and poor engagement with the intervention itself [1-3,10]. Alkhaldi and colleagues in a recent systematic review concluded that more research was needed in this area in order to be able to understand engagement with online interventions and trials [2]. On the other hand, the Internet has demonstrated potential in facilitating research into sensitive issues [11]. For instance, Web-based methods have been used in studies evaluating digital interventions for sexual health [12,13], problem drinking [14,15], smoking cessation [16], drug use [17], and mental health problems [18]. In these contexts, the anonymity offered by the Internet may encourage participants to take part where they otherwise would not [10], potentially counteracting some of the challenges mentioned earlier. This is not to say that Web-based trials of interventions for sensitive issues are not without pitfalls.

In the context of domestic violence (DV), very little is known about the potential to deliver interventions via the Internet, or about safely recruiting research participants to a Web-based randomized controlled trial [19], although several authors have examined these issues in face-to-face DV studies [20-22]. DV is characterized by a systematic pattern of physical, emotional, sexual, or financial abuse by one intimate partner toward another with the intent to intimidate and control [23]. It is overwhelmingly perpetrated by men toward women [23] and can lead to a range of negative health outcomes, serious injury, or death. Research into DV is typically fraught with methodological and ethical issues [24]. Many abused women will not identify themselves as someone who is experiencing DV, thereby presenting a challenge to researchers seeking to recruit them [11]. There are also potential safety issues (eg, loss of confidentiality, retaliation from an abusive partner or ex-partner) involved with engaging abused women [25,26]. Once recruited into a study, women can potentially be re-traumatized by insensitive or inappropriate questioning [26,27], and researchers must be mindful of the ways in which controlling perpetrators may monitor women’s movements or correspondence [24]. Due to the increased level of anonymity and privacy, as well as the ability to be accessed from anywhere, the Internet has the potential to overcome some of these issues [11], while also presenting new challenges that need to be attended to in the study design phase. This paper will use the case study of I-DECIDE, a Web-based healthy relationship tool and safety decision aid for Australian women experiencing DV [11,28], developed by the authors to illustrate how a randomized controlled trial of an intervention can be safely conducted via the Internet in this sensitive area [19]. We will focus on ethical and methodological challenges that occur specifically in Web-based DV trials, rather than on Web-based trials more broadly, as this topic has been explored in detail by others [3]. We conclude by presenting some recommendations for other researchers seeking to conduct Web-based research in this challenging and sensitive area.

### The I-DECIDE Study

The I-DECIDE website is described in detail elsewhere [11], as is the study protocol for the randomized controlled trial that was used to evaluate it (ACTRN12614001306606) [28]. In brief, the intervention contained interactive modules designed to guide women through a process of self-reflection and self-management. The modules focused on healthy relationships, safety/risk and priorities, and incorporated elements of motivational interviewing [29] and nondirective problem solving [30]. The culmination of these activities was an “action plan” of strategies and resources that was individualized to women’s priorities, relationship choices, and level of risk. The website was designed to be worked through once in a linear fashion (although different pathways through the website were possible depending on women’s answers to certain key questions). Women were not required to complete the entire website in one sitting; however once they had completed the session, they were not able to return to change their answers. It was hypothesized that working through the intervention modules would increase women’s levels of awareness about abuse occurring in their relationships, improve their self-efficacy, and enhance their sense of being supported.

The I-DECIDE website was evaluated through a pragmatic randomized controlled trial, comparing it with a website representing usual care (in this case, “usual care” was standard information on DV, a static emergency safety plan, and a list of general resources for DV). The trial was conducted almost entirely via the Internet. There were 422 women participants aged 16-50 years, who experienced fear of a current or ex-partner or any form of abuse from a current or ex-partner in the 6 months before recruitment. For both intervention and comparison groups, the study measures appeared after the informed consent and at the beginning of the website session, after which women were presented with either the intervention or comparison modules. Women who completed the baseline study measures (irrespective of whether or not they went on to complete the intervention or comparison modules) were followed up at 6 and 12 months via email prompts reminding them to log back in to the website. During follow-up visits, women completed the study measures and were given the option of going through the intervention or comparison modules a second time. Women were also able to log back in to the website at any time in between scheduled visits to access their action plan. Data collection was completed recently, with 80% of the baseline sample completing their 12-month follow-up visit. No adverse events (in this context, “adverse events” would include a woman being placed at increased risk of harm from her partner or experiencing extreme emotional distress) have been reported, and posttrial qualitative feedback from women via an open text box on the website and process evaluation interviews was overwhelmingly positive.

### Ethical Issues

As with all DV research, the I-DECIDE study required a strong and carefully thought-out ethical framework to ensure the safety and well-being of its participants. Human Research Ethics Committees (HRECs) in Australia (Institutional Review Boards in the United States) have a critical role in ensuring that studies adhere to the National Health and Medical Research Council’s code of conduct and respect the values of beneficence and nonmaleficence [31]. However, it has been suggested that ethics committees, which tend to operate from a predominantly biomedical framework, can find research involving so-called “vulnerable participants” challenging [26,32]. It has been suggested that they sometimes hold research teams to higher standards of ethical rigor in violence-related projects than other public health projects [32,33] and play a “gatekeeper“ role toward research participants [26,34], which can make it more difficult for researchers to gain approval for work in this area. Furthermore, Downes and colleagues in United Kingdom [32] have argued that the paternalistic attitude sometimes taken by HRECs undermines the decision-making capacity and agency of abused women to participate in research. They point out that all research has the potential to cause distress to a participant, even if the topic seems innocuous, and that women experiencing violence ought not to be prevented from participating simply because they may become upset. In fact, as Valpied et al [27] found in their qualitative exploration of abused women’s experiences taking part in a randomized controlled trial of a general practice counseling intervention, the benefits of research participation—such as a sense of empowerment, catharsis and self-awareness—generally far outweigh any short-term distress.

In the case of I-DECIDE, there were not only considerations surrounding women’s safety and well-being, but also ethical challenges unique to Web-based research [35,36]. For instance, determining whether and how a participant can provide informed consent via the Internet [13]; a lack of face-to-face contact between researcher and participant; determining whether data will be stored securely once collected; and how privacy can be protected [36] are the common concerns with regard to the Internet or social media research. A number of specific ethical issues arose in the I-DECIDE study where the context of DV intersected with the challenges of Web-based research. These are outlined below, along with how they were overcome.

### Protecting Women on the Internet

In face-to-face studies, DV researchers can use a number of strategies in order to ensure the safety and well-being of participants [26,37]. For example, using trained interviewers to speak with women so that they can respond appropriately to any signs of distress [38], ensuring that women are contacted at a time when the perpetrator is not present and taking measures to ensure that her participation in the study remains confidential. When a study is conducted via the Internet, however, many of these safeguards cannot be monitored, and new safety strategies need to be put into place to protect participants.

For I-DECIDE, many of our strategies focused on equipping women to protect their own safety on the Internet, since the team did not connect with them via the web, by telephone, or in person unless they needed to be validated or had a technical issue. Several of these strategies also relate to how the intervention would be delivered in a “real world” setting on completion of the trial. Before signing up to the study, women were provided with browser-specific instructions on how to clear their search history and use an “incognito” or “private” session. They were also advised to use a “safe” email address that the perpetrator could not access and were directed to set up a new email account through Gmail or Yahoo if they were uncertain whether their existing account was secure. As an additional strategy, all email communication about I-DECIDE referred to a “Women’s Health Study,” without any reference to DV, and emails were sent from a specific women’s health account to avoid any connection to DV or DV services.

The website itself was equipped with a “quick exit” bar positioned along the top of every screen. This enabled a woman to exit the website with a single click anywhere in the designated area should a perpetrator come up behind her while using I-DECIDE. On clicking the “quick exit” button, the existing browser window redirected to a generic weather website and further, a new browser tab opened up displaying the Google search engine. The data already inputted by the participant were saved, so that the next time she logged in to I-DECIDE she could continue where she left off. The landing page of I-DECIDE, which was publicly accessible, did not contain any information about DV or contain any images related to abuse. Rather, the website was identified as a “Women’s Wellbeing Project” and asked the following:

Do you worry about whether your relationship is healthy? Do you sometimes wonder if you are safe? If you are a woman aged between 16 and 50 and you have experienced relationship issues over the last 6 months you are invited to take part in this project.

Although a list of key DV support services were provided on the landing page, they were mixed with other women’s health resources (eg, for depression and smoking) to disguise the focus of the website. Beyond the landing page, the website’s content was protected through a randomly generated username and password that was sent to women’s safe email address upon signing up.

### Duty of Care in a Web-Based Trial

A major consideration for researchers in the DV area is ensuring that they fulfill their duty of care toward the women involved in a study [26]. In a face-to-face study, this would involve checking with participants regularly to ensure that they still consent to take part and that their safety and well-being is not being compromised [37]. If a researcher is alerted to a woman being in immediate danger, their duty of care obliges them to discuss safety options such as contacting police or DV crisis line [32,38] and compels them to alert authorities if a child is suspected to be in danger [39]. The anonymity of the Internet, however, requires new strategies to be developed to maximize women’s safety and well-being [40].

In the I-DECIDE study, although we could not determine with any certainty whether women taking part in the study were at immediate risk, we used a number of approaches to maximize their safety during engagement with the Web-based tool. As part of the intervention module, for instance, women were asked to complete the Danger Assessment [41], which measures her level of risk for severe violence and homicide, and the Composite Abuse Scale [42], which identifies abusive behaviors in a relationship. Their responses to these validated tools were scored and categorized by the I-DECIDE program, and matching messages were immediately provided on the screen for the woman to review. Any woman whose responses indicated a higher level of risk were provided with feedback advising them of this and suggesting they think about contacting the police. At the end of the website, these women first received an emergency safety plan (including strategies such as collecting important documents and setting up an emergency code word with a friend or family member if she was in distress) before an action plan tailored to their individual life priorities. Women at lower levels of risk received only the tailored action plan (although they could also access the emergency safety plan if needed). All women in the comparison group automatically received only the emergency safety plan.

To minimize the likelihood of women becoming distressed while participating in the study, the I-DECIDE website included supportive messaging and feedback at critical points. In particular, the messaging in response to the woman’s levels of danger and abuse was developed very carefully in consultation with community DV services. The messaging needed to communicate the woman’s level of risk without frightening or traumatizing her. The example below is the message for the highest level of risk.

You are experiencing some extremely dangerous things in your relationship. It's critical that you talk to someone you can trust, like a friend, family member, your GP (general practitioner/family doctor), or the police, about what is going on in your relationship. A bit later, this website will suggest some things you could try to help you increase your safety, and also to help take care of yourself during this stressful time. It will also provide you with details of some services (confidential and free) that can help you if you need. There is a national counselling hotline 1800-737-732 that you can call anonymously for advice, but for an emergency situation, call 000.

As an additional precaution, a study phone number was set up in case women needed to contact the researchers, despite the trial being otherwise carried out entirely via the Web [19,28]. A distress protocol was developed and distributed to all project team members. The protocol included active listening and exploring with the individual whether they had someone (friend, family, or service provider) trusted that they could talk to, who would understand and be supportive. Ultimately, very few women utilized the phone number, and those who did were enquiring about study participant gift vouchers or experiencing technical difficulties.

An adapted version of the validated Consequences of Screening Tool (COST) questionnaire [43] was embedded within both arms of the I-DECIDE website to ensure that the benefits of participation outweighed the harms for women. This tool asks women about how participation affects their feelings about themselves and their relationship. The adapted COST contained 10 items, each on a five-point Likert scale that indicated harm, benefit, or neutral options. On completion of baseline data collection, the harm/benefit data were reviewed by the researchers and the independent study Data Monitoring Committee before commencement of the 6-month follow-up. Although 10.9% (37/339) of women reported that their partner or ex-partner was aware that they were answering questions about DV, at follow-up many (62%, 23/37) reported a positive outcome from this—a finding consistent with previous DV research using this tool [44]. Additionally, almost all the women (93.5%, 317/339) reported that they were glad to be a participant in the I-DECIDE project, despite 11.5% (39/339) indicating that questions in the website had made them feel that their relationship problems were their own fault and 5.3% (18/339) feeling somewhat worse about themselves as a person. Research suggests that these negative emotions are common, but usually transient [27]. For instance, Valpied and colleagues [27], who analyzed data from the COST Questionnaire in a face-to-face DV intervention trial [44], suggest that women might feel worse about themselves because they realize that an abusive relationship is unsalvageable despite how hard they have tried to make it work. They argue that this is not necessarily a negative outcome, as it could lead to taking action for safety and well-being. Feeling at fault, or otherwise bad about oneself is also not necessarily a reflection on delivering the intervention or carrying out a trial via the web. In fact, the average score out of 10 from the I-DECIDE study sample regarding how supported they felt by the website was 9.

### Balancing Participant Safety With Study Visibility

Attracting over 400 women nationally to the I-DECIDE study involved a mix of recruitment strategies. In particular, the essential role of social media as a key part of our recruitment became apparent very early on in baseline data collection.

Colleagues in the United States [45], who developed the original “IRIS” safety decision aid website on which I-DECIDE is based [11] had enormous success using the free classified advertisements website, Craigslist, to attract women to their study. In New Zealand [19,46], where another version of the website underwent trial, the overwhelming majority of the sample came from ads on TradeMe, a trading post style website. In Australia, however, Craigslist is not widely used, and our ads were not successful in attracting any women to the study. Similarly, ads placed on Australian trading post website, Gumtree attracted less than 10 women to the study, despite paying for our ads to be prioritized in search listings.

As the most widely used social media platform in Australia [7] and one that has demonstrated success with other Web-based trials [47] and with hard-to-reach populations [48], Facebook was next identified as a potential source of recruitment. However, there are risks associated with the format of Facebook which needed to be overcome for a DV study. Facebook enables its users to present themselves in a user profile, accumulate “friends” who can post comments on each other’s pages, and view each other’s profiles. Facebook members can also join virtual groups based on common interests [49]. Facebook users can “like” or “share” content that then appears on their “newsfeed,” and can “follow” updates from pages associated with organizations. Although the option of setting up a page for the I-DECIDE study clearly had potential to facilitate the wide distribution of our ads, it also meant that the ad might appear on the newsfeed of an abused woman who otherwise had no DV-related content. Instead, two approaches were used. First, a paid advertisement that appeared on the side of screen for women in the target demographic (as this appeared alongside a range of other advertisements, this was deemed less risky). Second, we sent requests to Australian DV services and a range of other women’s health, fashion, and motherhood organizations to post our ad. Although this would still result in the ad appearing in women’s newsfeed, the women would already have been following these pages in order to receive the update, and we therefore were not adding to their existing level of risk. For all ads, the accompanying text “Please open the link in a new browser window” and “Share only if safe to do so” was included. Ultimately, around 60% of our sample was recruited using Facebook.

## Methodological Issues

In the I-DECIDE study, women were recruited, enrolled, consented, and randomized entirely via the web. As Murray and colleagues have noted [3], Web-based trials can experience a range of methodological issues including an unrepresentative sample, poor retention rates, and an inability to validate participants leading to multiple registrations or inclusion of ineligible participants. In the DV context, these issues, as well as others unique to the field, were experienced as part of the I-DECIDE study as outlined below.

### Ensuring That Participants Are Who They Say They Are

The I-DECIDE study was open only to women residing in Australia aged 16-50 years who had experienced fear of a partner or some form of abuse over the past 6 months. However, in theory, to sign up to the study a person needed only to click “Be A Part of the Project” from the publicly accessible landing page to commence the enrolment process. We were therefore presented with a challenge around how to prevent enrolment by ineligible women, “trolls,” or perpetrators seeking to disrupt the study.

Koziol-McLain and colleagues in New Zealand [46] asked women to input their full name and residential address upon signing up to their study. This was then compared automatically with the New Zealand electoral roll, which enabled them to check the following (1) the participant was female and (2) the participant was a resident of New Zealand. We initially endeavored to copy this approach; however, privacy laws in Australia regarding distribution of the entire electoral roll are extremely stringent, and we were unable to gain access to this document. Instead, a research team member manually validated each individual participant through the Australian Electoral Roll website. Any participant not able to be validated through their residential address was validated via email contact, social media, or a telephone call, as a last resort. This process was time consuming, but worthwhile to ensure that our sample did not contain bogus participants.

In terms of validating the age of participants, they were asked directly on sign up, “Are you aged between 16 and 50 years.” Women who answered “no” were exited from the enrolment process with a message explaining that study was limited to this demographic. To further cross-check participant age, women were asked for their date of birth once they commenced the demographic questions on the first screen of I-DECIDE. Although this approach would not have stopped the determined participants outside our target age range from lying about their age, it likely minimized the incidences of ineligible participants taking part.

It was anticipated that abused women might have concerns about providing information such as their residential address and contact telephone number. We therefore included a short explanation about why we were requesting these details and what would be done with the information:

*We need to collect your full name and a valid residential address.*

There are three reasons for this:

*To keep you safe;*

To ensure that no fraudulent participants (including men) can access the website;

*To send you a gift card to thank you for your participation.*

We will validate your details against the Australian Electoral Roll and will not use your details for any other purpose.

The details of the participants were recorded in a separate database from their responses to the study measures and the rest of the intervention. Although providing a name did mean that women were not anonymous, it was considered to be an acceptable trade off in order to deter bogus participants.

### Engaging Women Not Ready to Identify as ‘‘DV Victims”

A major consideration for the I-DECIDE study was how to include women who may not have been ready to acknowledge that their relationship was abusive [11]. As Bender [21] noted, recruiting women through specific locations such as shelters, courtrooms, or health clinics can be problematic, as levels of violence reported from women in these settings may not be representative of the overall population. Similarly, accessing women only through DV services and the community sector would have resulted in a particular demographic of women who were already seeking help for violence in their relationships and the project was interested in reaching women who may not have been aware of or previously accessed DV services. To overcome this challenge, we contacted a wide range of organizations and individuals likely to have a large number of female followers and asked them to promote the study. As mentioned earlier, strategies included not only social media posts about the study (eg, Facebook and Twitter), but also mentions in organizational newsletters, advertisements on blogs, and media interviews with members of the research team. Another successful source of recruitment was university student portals, provided that we were able to satisfy the relevant “gatekeepers.”

**Figure 1:**
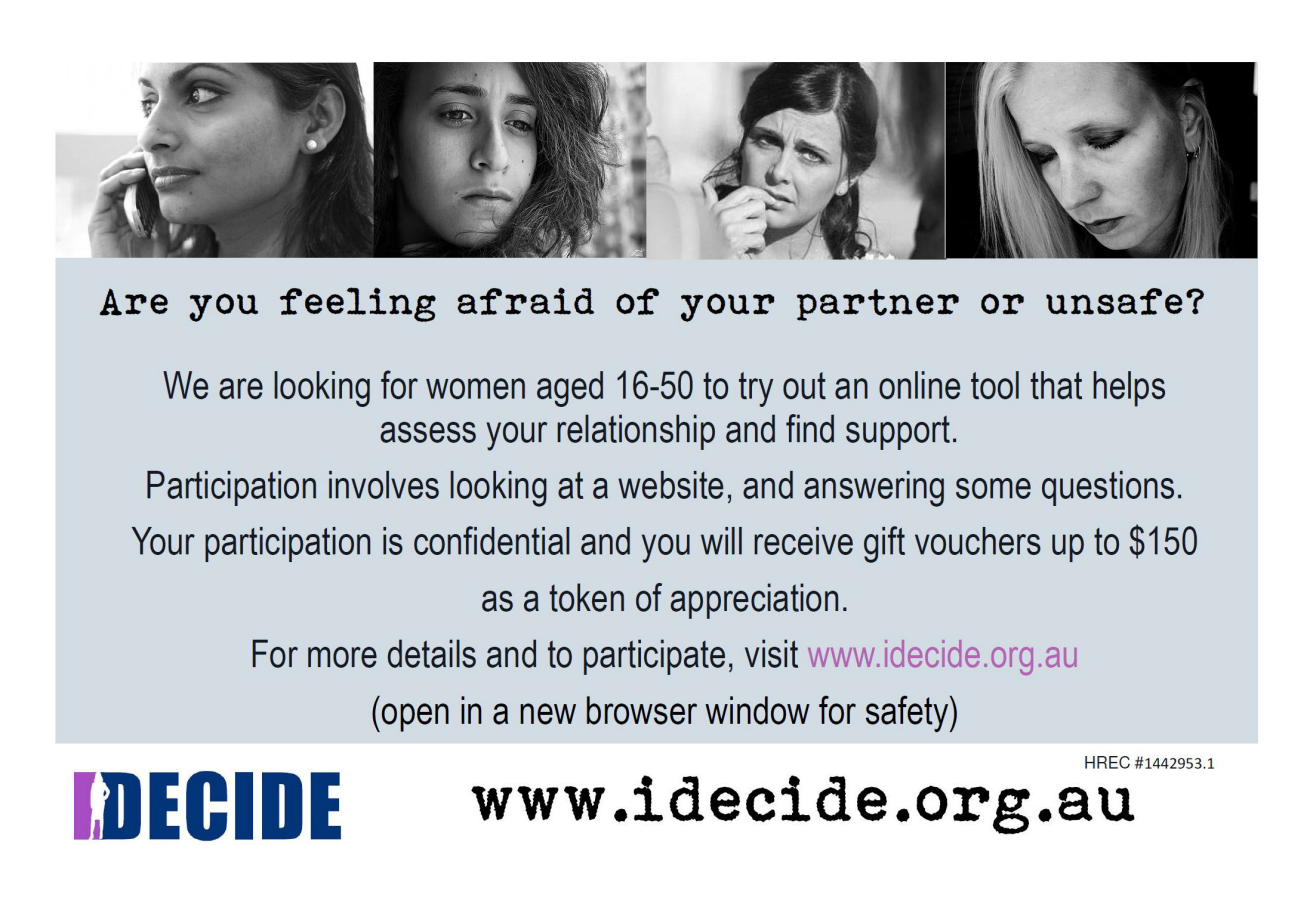
Study advertisement for I-DECIDE.

It is not immediately obvious how one might go about recruiting women experiencing DV without mentioning “violence” or “abuse,” while at the same time not misleading women as to the nature of the study. As a compromise, we referred to “relationship safety” or “feeling afraid of a partner” in all our recruitment materials (Figure 1). This was deemed to convey the topic without being too confronting or challenging for women in an earlier stage of awareness or readiness for action [50]. Although we did not ask women directly whether they perceived their relationship as violent, the study sample includes women of diverse ages and backgrounds who had experienced different types of abuse (Table 1). More detailed participant demographics will be reported at a later date.

**Table 1:** Brief I-DECIDE study demographics.

Study demographics		Mean (SD) or n (%)
Age in years (n=422), mean (SD)		33.74 (SD 8.48)
Aboriginal and/or Torres Strait Islander (n=376), n (%)		40 (10.64)
**Type of Abuse at 12 months (n=331), n (%)**		
	Emotional abuse (with or without harassment)	108 (32.7)
	Physical abuse and emotional abuse (with or without harassment)	36 (10.91)
	Severe combined abuse	125 (37.88)
	Not positive for abuse on Composite Abuse Scale [42]	61 (18.48)

### Creating Trust in the Absence of Face-to-Face Contact

Building rapport with research participants can be an important element of DV research that occurs in a face-to-face setting [26]. In an online setting, however, creating an impression of trustworthiness is much more difficult. In preliminary focus groups conducted during the design phase of the study, women stated that a professional design and the use of the university’s logo could help to distinguish the site from unreliable information on the Internet. Similarly, the language and the tone used throughout the website were designed to sound like an “empathetic expert,” such as a health professional or a counselor. The aim was to make women feel supported, empowered, and listened to, without being authoritative.

As another trust-building strategy in DV research, Logan and colleagues have emphasized the importance of engaging community partners [20]. This not only suggests to potential participants that the study has their best interests at heart, but also provides a source of referral for face-to-face support should they require it. To this end, we collaborated with several DV services and sought their feedback during the design phase of the website. Facebook posts by our partner organizations as well as by other high-profile DV organizations endorsing the study were successful at attracting women to click through to the I-DECIDE website.

### Encouraging Retention Over the Long Term

Retention of participants in all trials is often challenging, but Web-based trials tend to have most issues with the follow up [3]. When conducting research with abused women, there are additional challenges to contend with. For instance, women are often forced to move house or change their email address or telephone number if they are concerned for their safety or that of their children [51], which increases the likelihood they will be lost to follow up. To combat this, women were asked to provide the first name and either a telephone number or email address of one or two “safe contacts” on signing up to the study. It was explained to women that these contacts were needed only to stay in touch with them during the study.

*In case you move house or we lose touch with you for any reason during the trial, please put in the details of two trusted people we can contact to get in touch with you. Try to think of people who are unlikely to change their details over the next 12 months (for example, a parent or relative). We will only reveal that you are participating in a ‘women’s health study’.*

As an additional retention strategy, incentives were offered in increasing value for baseline, 6 month, and 12-month follow-up visits, with a maximum of $150 for completing all 3 visits. Researchers in the DV area consistently emphasize the importance of incentives for women as a way of recognizing their contribution [20]. Although women are often motivated to participate because of other, nonfinancial reasons (for instance, a desire to help other women [26]), it is generally considered good practice to offer them as a token of appreciation for their time and expertise. For the I-DECIDE study, incentives were offered in the form of electronic gift vouchers to a large national chain of stores.

Finally, although we had intended to use solely electronic means of communication to follow up women for 6- and 12-month visits, this was ultimately not successful. Initially, our study protocol dictated that the study tracking database would send out 3 automatic email reminders at various intervals after a woman’s session fell due, followed by an SMS reminder. Response rates remained lower than expected, and we therefore added a phone call reminder by a trained, study research assistant. Although the use of a research assistant can be seen as an “intervention” [40] and not indicative of how the website is likely to be accessed in a real world setting, our experience is that without this contact, it would be difficult to engage women, given the dynamics of an abusive relationship, in follow-up visits after a 6-month period of time.

### Conclusions and Implications for Future Research

This paper highlights the key ethical and methodological challenges involved with conducting a Web-based trial of a DV intervention. It highlights how the particular context of DV intersects with the constraints and opportunities of digital technologies. Our I-DECIDE case study demonstrates that trials of DV interventions can be conducted via the web, and women can be safely recruited, engaged in an intervention, and successfully retained over a 12-month period using almost exclusively Web-based strategies. Based on the lessons learned from our trial, we suggest the following recommendations for other researchers seeking to conduct trials of DV interventions using the Internet:

Publish ethical and “lessons learned” papers in conjunction with trials, so that ethics committees and other researchers can draw on this knowledge base to make informed decisions about future studies;

Ensure that the language used throughout Web-based interventions and the trial processes surrounding them is supportive and non-judgmental. Interventions should be developed in close consultation with victims/survivors and services;

Equip participants with information about Internet safety so that they can take charge of their own online footprint;

Explain why personal information is being collected;

Consider avenues of recruitment outside traditional DV services, and ensure that language is inclusive of women who may not be ready to name the abuse; and

Consider telephone contact with participants as a last resort to encourage retention in a trial over the long term.

## Abbreviations

DV: domestic violence
COST: Consequences of Screening Tool
HRECs: Human Research Ethics Committees
